# *BIRC6 (APOLLON)* is down-regulated in acute myeloid leukemia and its knockdown attenuates neutrophil differentiation

**DOI:** 10.1186/2162-3619-1-25

**Published:** 2012-09-04

**Authors:** Anna M Schläfli, Bruce E Torbett, Martin F Fey, Mario P Tschan

**Affiliations:** 1 Experimental Oncology/Hematology, Department of Clinical Research, University of Bern, Murtenstrasse 35, CH-3010, Bern, Switzerland; 2 Department of Molecular and Experimental Medicine, The Scripps Research Institute, La Jolla, CA, USA; 3 Department of Medical Oncology, Inselspital, Bern University Hospital, Bern, Switzerland

**Keywords:** *BIRC6*, Acute myeloid leukemia, Acute promyelocytic leukemia, ATRA, Neutrophil differentiation

## Abstract

**Background:**

Inhibitors of apoptosis (IAPs) were intensively investigated in the context of cancer where they promote tumor growth and chemoresistence. Overexpression of the IAP *BIRC6* is associated with unfavorable clinical features and negatively impacts relapse-free survival in childhood acute myeloid leukemia (AML). Currently, *BIRC6* levels in adult primary AML have not been compared to the expression in normal myeloid cells. Thus, we compared for the first time *BIRC6* levels in adult primary AML patient samples to normal myeloid cells and studied its regulation and function during neutrophil differentiation.

**Findings:**

We found significantly lower *BIRC6* levels in particular AML subtypes as compared to granulocytes from healthy donors. The lowest BIRC6 expression was found in CD34^+^ progenitor cells. Moreover, *BIRC6* expression significantly increased during neutrophil differentiation of AML cell lines and knocking down *BIRC6* in NB4 acute promyelocytic leukemia (APL) cells significantly impaired neutrophil differentiation, but not cell viability.

**Conclusion:**

Together, we found an association of low *BIRC6* levels with an immature myeloid phenotype and describe a function for *BIRC6* in neutrophil differentiation of APL cells.

## Introduction

*BIRC6 (a.k.a. APOLLON, BRUCE)* is an exceptionally large protein of 528 kDa belonging to the family of inhibitor of apoptosis (IAP). *BIRC6* contains one bacalovirus IAP repeat (BIR) domain that shows homology to the IAP *Survivin*. Furthermore, *BIRC6* is the only IAP with an ubiquitin-conjugating domain further pointing to a particular function of this protein in the IAP family [1]. Several groups reported that *BIRC6* executes its function via inhibition of *Smac* and *Caspase-9*[2,3]. Moreover, a study in breast cancer cells revealed that *BIRC6* inhibits cell death by *p53* destabilization and inactivation of *caspase-3*[4]. The role of *p53*-dependent *BIRC6* effector functions was confirmed by investigations of Ren et al. in mice and human lung cancer cells [5].

Due to their anti-apoptotic function it was hypothesized that overexpression of IAPs might contribute to tumorigenesis. *Survivin*, for example, one of the best studied IAPs is up regulated in most tumors and has been associated with their chemoresistence [6,7]. Recently, Houdt et al. [8] observed *BIRC6* overexpression in colon cancer stem cells when compared to more differentiated tumor cells. *BIRC6* expression protected colon cancer stem cells from the cytotoxic effects of oxaliplatin and cisplatin. Furthermore, knocking-down *BIRC6* led to growth inhibition in several cancer cell lines and xenografted mice and rendered the tumor cells more sensitive to 5-fluoruracil treatment *in vivo* and *in vitro*[9]. The significance of IAPs in the pathology of hematological malignancies however remains poorly understood and controversial data regarding an effect on prognosis were published. Carter et al. [10] did not find any prognostic significance for *Survivin* or *XIAP* expression in adult AML samples, whereas *BIRC6* overexpression is associated with unfavorable clinical features and negatively impacts on the 3-year relapse-free survival in childhood acute myeloid leukemia (AML) [11]. Similar results were obtained by Ismail et al. in childhood AML and acute lymphoblastic leukemia (ALL) [12]. Abe et al. [13] found lower expression of BIRC6 in bone marrow-derived cells of healthy donors compared to *de novo* AML samples.

To our knowledge, *BIRC6* levels in primary AML have never been compared to the respective expression in normal myeloid cells. Thus, we aimed at comparing *BIRC6* levels in a large cohort (n = 98) of adult AML patient samples and mature neutrophils from healthy donors. Since a hallmark of AML is a differentiation block of hematopoietic precursors at different developmental stages and since this block can be overcome by treating acute promylocytic leukemia (APL) patients with all-trans retinoic acid (ATRA) and low dose chemotherapy, we also analyzed the role of *BIRC6* during neutrophil differentiation of AML cells.

## Materials and methods

### Primary patient samples

A cohort of 98 samples from patients with a diagnosis of primary AML (FAB M0-M4) were enrolled on HOVON/SAKK (Dutch-Belgian Hematology-Oncology/ Swiss Group for Clinical Cancer Research Cooperative group) protocols −04, -04A, -29, and −42 (available at http://www.hovon.nl) between 1987 and 2006 [14-18]). All patients provided written informed consent in accordance with the Declaration of Helsinki. Patient data are summarized in the Table 1. *In vitro* differentiation of CD34^+^ progenitor cells was done as previously described [19].

**Table 1:** AML patient characteristics from the HOVON/SAKK cohort

		**Patient characteristics**			**FAB classification**					**Cytogenetics**				
**Cohort**	**Variables**	Age (y)	Sex (female/male)	**Total**	M0	M1	M2	M3	M4	t(8;21)	inv (16)	t(15;17)	CK	NK
HOVON/ SAKK	**Range**	17-74	-	**-**										
	**Mean/median** **/ %**	43.09/43.00 (mean/median)	59.18/40.82	**100**	4.08	16.33	32.65	19.39	27.55	20.41	17.35	20.41	23.47	18.37
	**No. of patients**		58/40	**98**	4	16	32	19	27	20	17	20	23	18

*FAB*, French-American-British; *CK*, complex karyotype; *NK*, normal karyotype.

### Cell lines and culture conditions

The acute myeloid leukemia (AML) cell lines HL60, FAB M2, the ATRA-resistant subline HL60-R, NB4, FAB M3 (acute promyelocytic leukemia; APL), the ATRA-resistant subline NB4-R2 and HT93 were kept in RPIMI-1640 culture media (Sigma-Aldrich, Buchs, Schweiz) containing 10 % foetal bovine serum (FBS). In order to differentiate the AML cell lines towards granulocytes 1 μM *all*-trans retinoic acid (ATRA) was added to the cells that were seeded at a density of 0.2x10^6^ cells/ml. Successful neutrophil differentiation was assessed by CD11b surface and by increased granulocyte colony-stimulating factor receptor (*G-CSFR; CSF3R*) mRNA expression.

### Quantitative real-time RT-PCR (qPCR) and TaqMan Low Density Array (LDA)

For RNA extraction the miRCURY RNA Isolation Kit from Exiqon was used. RT-PCR has been described elsewhere [19]. Quantitative measurement of *BIRC6* and *G-CSFR* mRNA was performed using the TaqMan® Gene Expression assays Hs00212288_m1 and Hs00167918_m1, respectively (Applied Biosystems, Rotkreuz, Switzerland). LDA measurements as well as data analysis were done as described [20]. *HMBS* primers and probes have been described previously [21]. Measurements were carried out on an ABI PRISM 7500 Sequence Detection System (Applied Biosystems, Rotkreuz, Switzerland).

### Generation of *BIRC6* knock-down cell lines

NB4 cells were transduced with a lentiviral vector (pLKO.1) expressing a small hairpin (sh)RNA targeting the *BIRC6* mRNA (NM_016252.x-2281s1c1, Sigma-Aldrich, Buchs, Schweiz). Lentivirus production has been described previously [19]. As a non-targeting control we used the SHC002 lentiviral vector. Two days after transduction, NB4 cells were selected with 1.5ug/ml puromycin (Sigma-Aldrich, Buchs, Schweiz) for one week.

### Cell viability assay

For Trypan blue exclusion assay cells were washed with PBS before diluting 1:10 in 0.4 % Trypan blue solution (Sigma-Aldrich, Buchs, Switzerland). For AnnexinV staining 1x10^5^ cells were washed in 500 μl binding buffer (PBS with 0.33 g/L Ca^2+^) and resuspended in a final volume of 100 μl. 5 μl AnnexinV-PE were added (Immunotools, Germany) and the samples were incubated for 15 minutes at room temperature, before FACS analysis.

### Statistical analysis

Differences between two groups were assessed using the non-parametric Mann–Whitney-U test. P-values <0.05 were considered to be statistically significant.

## Results

### Repression of *BIRC6* mRNA in AML patients with particular chromosomal aberrations

**Figure 1:**
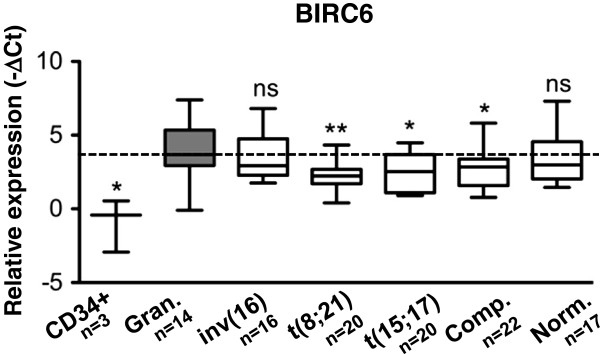
***BIRC6 *mRNA expression is significantly lower in particular AML subtypes as compared to granulocytes.** Relative expression levels of *BIRC6* mRNA were determined by qPCR analysis. Raw Ct values were normalized to *HMBS* and *ABL1* (−ΔCt) and represent log_2_ expression levels. *BIRC6* expression in AML subtypes FAB M0-M4 and the following molecular lesions: (inv(16), t(8;21), t(15;17), Comp. = Complex Karyotype, Norm. = Normal Karyotype). Relative *BIRC6* expression levels were compared to the relative expression in granulocytes from healthy donors (Gran.) and CD34^+^ progenitor cells. Mann–Whitney U test *p < 0.05, **p < 0.01, ns not significant.

To study the *BIRC6* expression patterns in normal versus leukemic myeloid cells, we measured *BIRC6* levels in a large cohort of 98 primary AML patients (FAB M0-M4), in 24 granulocyte preparations from healthy donors and in 3 CD34^+^ progenitor cell samples. We were able to detect *BIRC6* in 95/98 AML patient, in 14/24 granulocytes and in 3/3 CD34^+^ progenitor cell samples. Surprisingly, we found significantly lower *BIRC6* levels in AML patients with the translocations t(8;21) and t(15;17) as well as in AML patients with a complex karyotype, whereas no significant differences in *BIRC6* expression was found in AML patients with inv(16) or normal karyotype as compared to its expression levels in granulocytes from healthy donors (Figure 1). We found the lowest *BIRC6* mRNA levels in in CD34^+^ progenitor cells (Figure 1). Together, our data suggest an association of low *BIRC6* expression with an immature myeloid phenotype.

### Significant *BIRC6* inductions during ATRA-induced neutrophil differentiation

**Figure 2:**
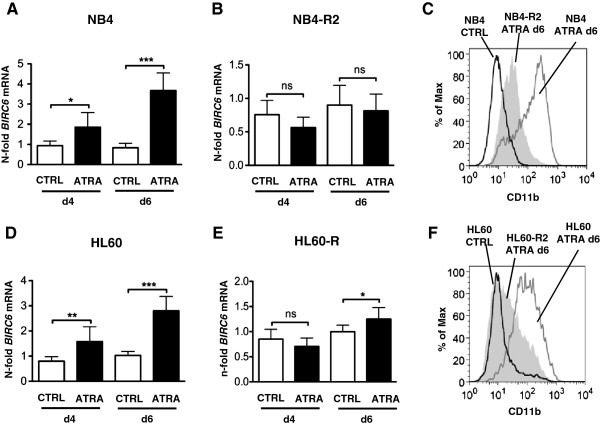
**Significant induction of* BIRC6 *mRNA levels upon neutrophil differentiation of AML cells.** (**A**) *BIRC6* qPCR analysis of NB4 APL cells treated with 1 μM ATRA for 4 and 6 days. Raw Ct values were normalized to *HMBS* and to the untreated control of day 4. Results from four independent experiments in duplicates are shown. (**B**) *BIRC6* qPCR analysis of ATRA-resistant NB4-R2 APL cells treated as in A. (**C**) *CD11b* surface expression of NB4 and NB4-R APL cells upon six days of ATRA-induced neutrophil differentiation. CD11b was measured by FACS analysis. (**D**) Neutrophil differentiation of HL60 AML cells with 1 μM ATRA for 4 and 6 days, respectively. *BIRC6* mRNA expression was assessed by qPCR as in A. (**E**) *BIRC6* qPCR analysis of ATRA-resistant HL60-R cells treated as in A. (**F**) The differentiation levels of HL60 and HL60-R were assessed by *CD11b* surface expression upon 6 days of ATRA-induced neutrophil differentiation. Mann–Whitney U test *p < 0.05, **p < 0.01, ***p < 0.001, ns not significant.

In order to assess a possible role of *BIRC6* in neutrophil differentiation of AML cells, we took advantage of several AML cells line models for neutrophil differentiation. First, we differentiated NB4 APL cells with ATRA towards neutrophils. In accordance with the low *BIRC6* expression in undifferentiated AML patients compared to mature neutrophils, *BIRC6* mRNA levels were up-regulated 1.8- and 3.6-fold at day 4 and 6 of ATRA treatment, respectively (Figure 2A). In order to exclude a direct effect of ATRA on *BIRC6* expression, we also treated ATRA-resistant NB4-R2 APL cells with ATRA. *BIRC6* transcripts were only marginally up-regulated in these cells further supporting a particular role of *BIRC6* in neutrophil differentiation (Figure 2B). Neutrophil differentiation of NB4 and NB4-R2 cells was assessed by CD11b surface expression (Figure 2C). A similar induction of *BIRC6* mRNA expression upon neutrophil differentiation was seen in HT93 APL cells (data not shown). In a further experiment, we determined *BIRC6* expression in HL60 and ATRA-resistant HL60-R AML cells during neutrophil differentiation. We observed a significant 1.6- and 2.8-fold increase of *BIRC6* transcript expression at day 4 and 6, respectively. No significant change in *BIRC6* expression was seen in HL60-R cells upon 4 days of ATRA treatment, whereas at day 6 a minor but significant increase was seen (Figure 2D and E). Neutrophil differentiation in HL60 cells was confirmed by CD11b FACS analysis (Figure 2F). ). Consistent with our data showing increased *BIRC6* expression upon neutrophil differentiation of NB4 and HL60 AML cells, we observed a 3.3- and 2.8-fold increase in *BIRC6* mRNA expression at day 3 and 6 upon *in vitro* granulocytic differentiation of CD34^+^ progenitor cells, respectively (data not shown).

Overall, our findings clearly show an association of increased *BIRC6* expression with neutrophil development of AML and CD34^+^ progenitor cells.

### Knocking down *BIRC6* attenuates neutrophil differentiation but not cell death and proliferation of APL cells

**Figure 3:**
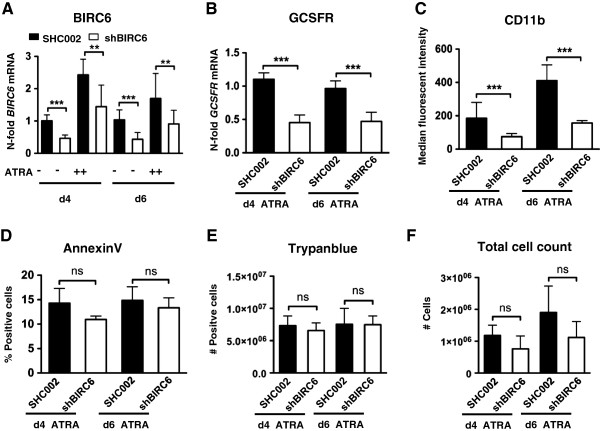
**Knocking-down* BIRC6 *attenuates neutrophil differentiation of APL cells.** (**A**) NB4 *BIRC6* knock-down cells (sh*BIRC6*) were generated by lentiviral transduction of shRNAs targeting the *BIRC6* mRNA. *BIRC6* knockdown efficiency compared to control transduced (SHC002) NB4 cells was determined by qPCR analysis. Values are shown as n-fold regulation compared to control transduced cells. (**B**,**C**) NB4 sh*BIRC6* and SHC002 were treated with 1 μM ATRA for 4 and 6 days, respectively. Neutrophil differentiation was determined by *G-CSFR* mRNA and *CD11b* surface expression. The raw Ct values of *G-CSFR* were normalized to *HMBS* and to the Ct values of the respective control transduced cells under ATRA treatment. *CD11b* expression is shown as mean fluorescence intensity (MFI). (**D**,**E**) Cell death in *BIRC6* knock-down cells at day 4 and 6 of ATRA treatment was assessed by AnnexinV staining and Trypanblue exclusion assays. Data are given as percentage or total number of death cells, respectively. (**F**) Total cell counts were determined using a hemocytometer. There was no difference in total cell number at day 4 or 6 of ATRA treatment between sh*BIRC6* or control NB4 cells. Mann–Whitney U test **p < 0.01, ***p < 0.001, ns not significant.

To test if specific *BIRC6* depletion inhibits neutrophil differentiation of APL cells, we generated NB4 *BIRC6* knockdown cells. *BIRC6* knockdown efficiency was confirmed in control and ATRA-treated sh*BIRC6* expressing NB4 cells (Figure 3A). Neutrophil development as determined by G-CSFR mRNA as well as CD11b surface expression was on average 50 % reduced compared to the non-targeting control transduced NB4 cells at day 4 of ATRA treatment (Figure 3B-C).

Since *BIRC6* is involved in the inhibition of apoptosis, we were asking if the observed lack of neutrophil differentiation in NB4 *BIRC6* knockdown cells is due to increased cell death in these cells. For this purpose we assessed cell death by Trypan blue exclusion and Annexin V flow cyotometry at day 4 and 6 of ATRA treatment. We did not find any significant differences in cell viability between NB4 control and *BIRC6* knockdown cells during ATRA-induced neutrophil differentiation (Figure 3D and E). Furthermore, control transduced as well as *BIRC6* knockdown NB4 cells showed no significant differences in total cell counts at day 4 and 6 of ATRA treatment (Figure 3F).

In summary, we showed that inhibiting *BIRC6* significantly affects neutrophil differentiation of APL cells, but not cell viability.

## Discussion

In this report we publish for the first time that AML patient samples with the t(8;21), the t(15;17) or a complex karyotype express BIRC6 significantly lower than normal human granulocytes. Subtype-specific BIRC6 expression in AML is supported by earlier findings published by Ismail et al. [12]. Low expression of the anti-apoptotic IAP *BIRC6* in AML may seem controversial given the often high expression of IAPs in cancer. Our findings of high *BIRC6* expression in granulocytes versus AML may reflect the cellular differentiation status of these cells rather than a cancer-associated deregulation. This hypothesis is confirmed by our observation that *BIRC6* mRNA levels are clearly reduced in CD34^+^ myeloid precursor cells and increase during granulocyte differentiation. These data are in line with previous findings showing that *BIRC6* is down-regulated in bone marrow-derived cells if compared to *de novo* AML samples [13]. Furthermore, the expression of the *BIRC6* related *XIAP* is induced upon monocyte differentiation and contributes to monocyte cell survival. In the same study, *XIAP* levels declined during neutrophil differentiation supporting the cell type specific regulation of IAPs in myeloid cells [22]. Consistent with the monocyte specific expression of *XIAP*, a correlation of *XIAP* levels with monocytic markers in AML was found [23]. In contrast to these finding in adult AML, in childhood *de novo* acute myeloid leukemia the levels of *XIAP* correlated with an immature FAB-subtype [24]. This may suggest different *XIAP* functions in distinct leukemic entities.

In conclusion, we were able to link increased *BIRC6* mRNA expression with neutrophil differentiation and inhibiting *BIRC6* resulted in attenuated neutrophil differentiation but did not alter cell survival. In summary, we established a new role for *BIRC6* in neutrophil differentiation of AML cells.

## Abbreviations

IAP, Inhibitor of apoptosis; AML, Acute myeloid leukemia; APL, Acute promyelocytic leukemia; ATRA, All-trans retinoic acid; BIR, Bacalovirus IAP repeat; ALL, Acute lymphoblastic leukemia.

## Competing interest

No competing interest to be declared.

## Author`s contributions

AS performed the experimental research, interpreted the data and drafted the article. BET provided primary cells and essential reagents, analyzed patient data and revised the article. MFF investigated the initial concept and experimental design and revised the drafted article. MPT designed the project and gave final approval of the submitted manuscript. All authors read and approved the final manuscript.
